# PReS-FINAL-2058: Lipid peroxides, lipophuscin, sulfhydryl groups and TOS in children with JIA

**DOI:** 10.1186/1546-0096-11-S2-P70

**Published:** 2013-12-05

**Authors:** H Mazur Zielinska, M Zielinski, D Karbowska, L Pilarz, E Birkner

**Affiliations:** 1 Department of Pediatrics, Zabrze, Poland; 2 Medical University of Silesia, Zabrze, Poland; 3 Department of Biochemistry, Medical University of Silesia, Zabrze, Poland

## Introduction

The etiology of most joints' inflammations is unknown. Arthritis in children can have a diversified course. Various parameters useful in diagnosis and treatment of different forms of inflammations of the joints are being researched. Reactive oxygen species in large concentrations are toxic and cause, among others, the phenomenon of polyunsaturated fatty acids lipid peroxidation, which results in the formation of aldehyde compounds. Non enzymatic antioxidants include: lipid peroxides (LHP = LOOH), lipophuscin (LF). An important part of non enzymatic antioxidative defense are sulfhydryl groups-SH. Total oxidative status (TOS) is used as an indicator of the overall oxidative potential of cells.

## Objectives

The aim of the study was to find how the level of the selected oxidative parameters changes in serum in children with inflammation of the joints. The correlation between the selected oxidative parameters and disease's relapses is also studied.

## Methods

The studied parameters were measured in blood serum of 59 patients with JIA, aged from 2 to 18 years old, hospitalized in the Rheumatology Division of the Department of Pediatrics, Silesian Medical University. The control group consisted of 25 healthy children.

## Results

See Table 1.

**Table 1:** 

	JIA	Control group
SH	**246,7±53,81**	**329,8±24,23**

LF	**741,2±208,8**	**827,5±192,7**

LOOH	**49,44±27,14**	**25,24±18,83**

TOS	**56,81±45,01**	**47,89±24,28**

## Conclusion

The studied parameters of oxidative status differ between children with arthritis and healthy ones. Lipid peroxide levels are dependent on the type of arthritis. The LOOH and lipofuscin concentrations in healthy children as compared with a group of children with JIA differ. Higher values occur in the acute forms of JIA, but the difference is not statistically significant. There is no correlation in the total oxidative status (TOS) and the inflammation's level in the joints.

## Disclosure of interest

None declared.

